# Adaptive deep brain stimulation in Parkinson's disease

**DOI:** 10.1016/j.parkreldis.2015.09.028

**Published:** 2016-01

**Authors:** M. Beudel, P. Brown

**Affiliations:** a Department of Neurology, University Medical Centre Groningen, University of Groningen, Groningen, The Netherlands; b Nuffield Department of Clinical Neurosciences, John Radcliffe Hospital, University of Oxford, OX3 9DU, UK; c The Medical Research Council Brain Network Dynamics Unit at the University of Oxford, OX1 3TH, UK

**Keywords:** Deep brain stimulation, Parkinson's disease, Brain-computer interface

## Abstract

Although Deep Brain Stimulation (DBS) is an established treatment for Parkinson's disease (PD), there are still limitations in terms of effectivity, side-effects and battery consumption. One of the reasons for this may be that not only pathological but also physiological neural activity can be suppressed whilst stimulating. For this reason, adaptive DBS (aDBS), where stimulation is applied according to the level of pathological activity, might be advantageous. Initial studies of aDBS demonstrate effectiveness in PD, but there are still many questions to be answered before aDBS can be applied clinically. Here we discuss the feedback signals and stimulation algorithms involved in adaptive stimulation in PD and sketch a potential road-map towards clinical application.

## 1 Introduction

Deep Brain Stimulation (DBS) is one of the most effective treatments for advanced Parkinson's disease (PD). However, although it has been applied for over 25 years, there are still limitations in terms of efficacy, side-effects and efficiency. At present, conventional DBS targeted at the subthalamic nucleus (STN) or globus pallidus interna (GPi) provides, on average, only about 40% improvement in the motor items of the Unified Parkinson's Disease Rating Scale (UPDRS III) OFF dopaminergic medication. Furthermore, there is even evidence that DBS can, paradoxically, worsen motor functioning by not only influencing pathological but also physiological neural activity [1], [2]. Next to this, the potential of conventional DBS is often limited due to stimulation induced side-effects. Finally, the capacity of non-rechargeable batteries is limited, and many patients are unsuitable for rechargeable devices. Thus in some patients battery replacement surgery may need to take place every few years.

Although there are many stimulation parameters that can be adjusted, the key attribute of conventional DBS is that stimulation is delivered continuously, and is thus non-adaptive. In theory, DBS could work more effectively with less side effects and be more efficient were it only to stimulate as and when necessary. This type of stimulation is called adaptive DBS (aDBS).

For aDBS to be achieved, it must be subject to feedback control and adjustments automatised. In PD, there are a variety of measurements that could form the basis for feedback, particularly the spontaneous electrophysiological activity recorded in the brain, termed the local field potential (LFP), and accelerometer measurements of tremor activity. The requirements of these feedback signals are partly dictated by the precise means of stimulation. High frequency DBS just requires feedback signals to be indicative of current clinical state, but not necessarily causally important. However, as we will discuss, some stimulation patterns under development require the sensed signal to be causally important, as stimulation is specifically tailored to suppress the sensed signal.

## 2 Potential feedback signals in aDBS according to impairment in Parkinson's disease

### 2.1 Bradykinesia and rigidity

At present there is substantial experimental evidence that elevated beta (13–35 Hz) frequency band power in the STN or pallidal LFP is associated with bradykinesia and rigidity, but not tremor, in PD [3]. This beta signal is also robust, remaining recordable over many years [4]. More recently, functional distinctions have been suggested between beta oscillations in the low (13–20 Hz) and high (21–35 Hz) beta frequency range. Although combined magnetoencephalography and STN LFP recordings show cortical-subcortical coherence in the high beta range [5], STN LFP recordings demonstrate particular modulation of low beta power after the application of dopaminergic medication [6] or in the correlation with Parkinsonian severity in the untreated state [7]. It remains unclear whether the power of low or high frequency beta might serve as the better feedback signal for aDBS.

Besides the power of beta oscillations, the volatility of beta power might potentially serve as a biomarker as well. The reason for this is that coefficient of variation (CV) of beta power in the OFF-state is significantly, inversely, correlated with UPDRS III scores and with the change in UPDRS III scores after the application of dopaminergic medication [8]. Another feature of activity in the beta frequency band that has recently come to the fore is the modulation of other frequencies of LFP activity by the phase of the beta signal. This has been noted in several forms. At the level of the motor cortex phase amplitude coupling (PAC) involves modulation of the amplitude of cortical broad gamma oscillations (from ∼50 to 200 Hz) by the phase of beta oscillations in patients with PD. Such PAC decreases in relation to movements [9], and is decreased by DBS, with the degree of reduction correlating with motor improvement [10]. This cortical PAC has been suggested as another potential biomarker for aDBS [10].

Another form of PAC has been reported in the STN, and involves amplitude modulation of LFP activity in the range between 200 and 400 Hz, termed high frequency oscillations (HFO's), by the phase of STN beta activity [11]. The peak frequency of HFO's changes from around 250 Hz to about 340 Hz after treatment with dopaminergic medication, and the strength of HFO PAC correlates with the UPDRS III score OFF medication. However, it is not yet known which particular UPDRS III items best correlate and to what extent DBS influences HFO PAC, although electrodes that show greater HFO PAC turn out to be more likely the contacts that are clinically effective [11].

PAC provides an interesting potential feedback signal for aDBS, but also one that is challenging to record and analyse on-line, particularly given the very low amplitude of high frequency activities. Whether PAC it is more directly informative of clinical state than beta band power or its variation also remains to be seen. Nor is the causal relevance of any of these beta related phenomena established with respect to different Parkinsonian symptoms.

### 2.2 Tremor

Beta band LFP power does not correlate with tremor. Rather neural activity at tremor frequency (∼5 Hz) and its first harmonic (∼10 Hz) has been recorded in the cortico-basal ganglia-cortical loop in tremulous PD patients and its amplitude suggested as a possible feedback signal for aDBS [12]. It seems plausible that these central oscillations at tremor related frequencies might also be causally related to tremor, particularly as surgical lesioning or stimulation of key sites at which such oscillations have been recorded lead to tremor suppression. This opens up the possibility of aDBS based on phase-interference stimulation techniques (see below). Tremor is also easily recorded using peripheral accelerometers providing another potential source of feedback with which to modulate aDBS.

### 2.3 Dyskinesias

Although DBS generally affords dramatic amelioration of dyskinesias in PD, 2–4% of patients experience DBS induced dyskinesias. Spectral features in the LFP that have been associated with dyskinesias are a shift from elevated beta power to increased activity in the 4–10 Hz and/or 65–90 Hz ranges [13], [14]. Interestingly, the low-frequency spectral peak is also seen in the LFP power spectrum of dystonia patients recorded in GPi [15] and stimulation of the STN at 5 Hz has induced involuntary choreiform movements in PD patients undergoing DBS surgery [16]. In theory such shifts in LFP frequency could serve to denote dyskinesias, but these might be more faithfully captured and fed back from peripheral inertial sensors. Alternatively, it might be that by tracking only beta power, stimulation can be reduced when such power falls low, thereby avoiding DBS induced dyskinesias.

### 2.4 Freezing & other axial features

After 10–15 years, it is often not limb bradykinesia-rigidity, but axial motor features that dominate the motor phenotype of PD. Contrary to ‘appendicular’ motor signs, axial symptoms respond less well to STN or pallidal DBS. Lately, the pendunculopontine nucleus (PPN) has been suggested as a more successful target for the treatment of gait and balance problems [17]. A recent report showed decreased 5–12 Hz activation in the PPN when patients were unable to step because of severe freezing of gait [18]. Conversely, when patients on dopaminergic medication were able to walk, 5–12 Hz activity increased [19]. Could PPN LFP power over 5–12 Hz form the basis for aDBS in this nucleus? So far, however, no data have been presented on the modulation of local oscillatory activity by PPN DBS, and the efficacy of stimulation of this target is still debated.

## 3 Stimulation parameters for aDBS

Many stimulation parameters can be used in aDBS. In the aDBS studies that have been published up to now [20], [21], [22], [23], high frequency (∼130 Hz) stimulation, with regular pulses with a fixed inter-pulse interval, were given. There are two different approaches to the application of high frequency aDBS: a binary approach, with effective stimulation either on or off, and a scalar approach with stimulation voltage being varied up to and including therapeutic values. Care has to be taken with both approaches that stimulation voltage is not rapidly increased with the induction of paresthesia. This issue is particularly important with binary on-off stimulation, where it is managed by the incorporation of a ramping of stimulation onset and offset. With regard to the scalar stimulation approach, the value of stimulating at sub-threshold voltages remains to be clarified.

Recent findings suggest an alternative approach to stimulation when oscillatory activity is believed to be causally important. This opens up the possibility of aDBS based on phase-interference stimulation techniques. When the thalamus is stimulated at low frequencies shocks delivered at certain phases of the peripheral tremor, and hence presumably of central tremor oscillations, reinforce peripheral tremor whereas those delivered at other phases attenuate tremor. By steering aDBS to the latter phases, a very selective form of aDBS treatment could potentially be performed [24], [25]. Support for approaches in which oscillation phase is detected and then stimulation delivered to optimally disturb or cancel oscillations comes from two studies. The first will be described in the following section [26]. In the second study, a non-invasive technique, transcranial alternating current stimulation (TACS), was able to reduce PD tremor by delivering sinusoidal varying current at the correct frequency and phase offset to cancel central tremor oscillations [27].

## 4 Current experience of aDBS in non-human primates and patients with Parkinsonism

The first experimental evidence of the successful application of aDBS in Parkinsonism came from non-human primates [26]. In this landmark study two monkeys were implanted with electrodes in the GPi and ipsilateral primary motor cortex. The most successful stimulation regime tested involved detection of single neuron spike discharges in motor cortex and the subsequent delivery of series of seven pulses to the GPi at high frequency, after a delay of 80 ms. The delay of 80 ms coincided with the period of spontaneous oscillations in the BG-cortical loop in this model, and therefore the approach might be considered a form of phase interference of pathological oscillators. Bradykinesia improved significantly compared to 130 Hz (continuous) cDBS despite less overall stimulation.

The results of the first patient to be stimulated with aDBS were briefly reported in 2012 [20], and these authors followed this up with publication of a detailed series of 8 further patients in 2013 [21]. The approach taken was similar across these reports, and involved the triggering of unilateral, high frequency STN DBS whilst a threshold level of beta activity was exceeded at the stimulation target. Adaptive DBS was only tested for short periods of about 5 min, and was evaluated through blinded video assessments. Nevertheless, aDBS proved more effective than conventional continuous, ipsilateral, high frequency DBS despite stimulating for less than 50% of the time.

**Fig. 1:**
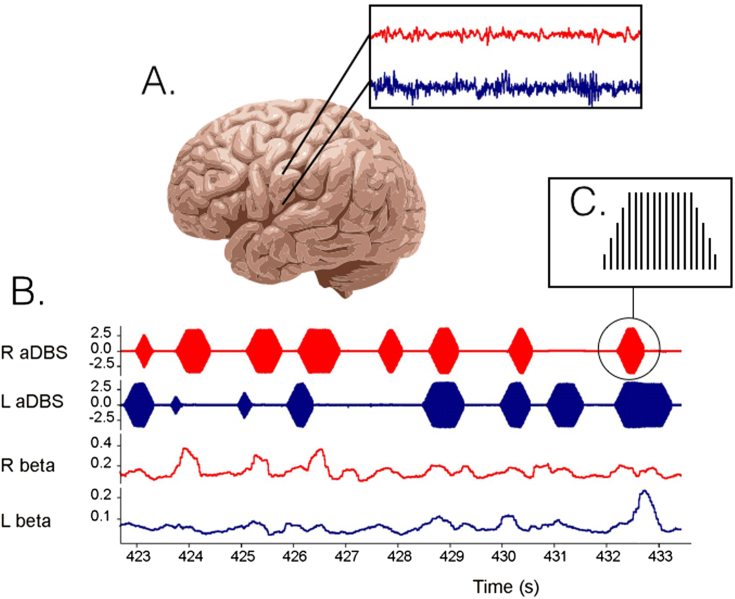
Example of bilateral adaptive DBS (aDBS) based on LFP beta oscillation power in the STN of both sides. A. LFP's are recorded from the non-stimulating DBS electrode contacts resulting in a left (blue) and right (red) LFP signal. B. After filtering around a patient specific beta peak, in this case 20 ± 3 Hz, its amplitude can be calculated real-time (lower two traces). When beta power exceeds a threshold, stimulation is delivered (upper two traces). C. In this example, high frequency (130 Hz) stimulation is provided with gradually increasing and decreasing voltage to limit stimulation induced paraesthesiae. The stimulation across the two sides is discontinuous and independent. (For interpretation of the references to colour in this figure legend, the reader is referred to the web version of this article.)

The above is encouraging, but aDBS was only applied unilaterally and for brief periods, so that axial signs and gait could not be assessed. Moreover, DBS-related side effects, such as speech impairment, are predominantly seen with bilateral stimulation. So far the effects of bilateral aDBS have been reported in a small series and in one case report. In the former, STN aDBS was delivered in four patients bilaterally with independent triggering of stimulation in the two hemispheres according to the amplitude of beta activity at the corresponding electrode [23] (Fig. 1). Blinded video assessments of both limb and axial features confirmed a 43% improvement compared to no stimulation, despite an average time on stimulation of only 45%. Although aDBS was not directly compared with conventional DBS, the degree of improvement was at least as good as in other studies where conventional, continuous stimulation was evaluated with blinded video assessments. Levodopa was well tolerated during aDBS and led to further reductions in the time on stimulation. The case report of bilateral aDBS is particularly tantalising as it provides the first suggestion that aDBS may reduce stimulation-induced side-effects given that less energy is delivered over time [22]. Both aDBS and cDBS improved axial symptoms, aDBS delivered for over 2 h improved bradykinesia better than cDBS, and critically dyskinesias were diminished with aDBS in the ON medication state.

One of the unexpected but consistent features of the above studies is that aDBS was more effective than conventional DBS in the same subjects [21], [22], [26]. This needs to be confirmed further, but raises the possibility that the benefits of suppressing pathological activity with conventional continuous DBS may be partly offset by the simultaneous interruption of residual periods of physiological motor processing [1]. Adaptive DBS, by sparing periods where the LFP suggests relative normality of functioning, may paradoxically then lead to improved efficacy.

In conclusion, clinical experience of aDBS in PD is relatively scant, and confined to the use of simple beta amplitude determined control of high frequency stimulation voltage and duration. Although the results are encouraging as proof-of-principle, DBS-related side-effects like speech and gait impairment remain to be objectively contrasted between aDBS and conventional DBS, and all the reports to date involve the testing of patients a few days after electrode implantation, when in some centres leads are still accessible before surgery to insert the implantable pulse generator. Unfortunately this period is confounded by the so-called stun effect [28], which means that parkinsonian deficits may be temporarily ameliorated and LFPs potentially unrepresentative of the chronic state. Although this could potentially be circumvented by performing these trials around the time of battery replacement, the time available then is short and ultimately aDBS will have to be trialled using a chronically implanted device.
